# Relationship between endotoxin core, staphylococcal and varicella antibody levels and outcome following aortic valve replacement surgery: a prospective observational study

**DOI:** 10.1186/s13741-018-0101-z

**Published:** 2018-09-20

**Authors:** Andrew Smith, Sarka Moravcova, Thomas A. Treibel, Patricia Colque-Navarro, Roland Mollby, James C. Moon, Colin Hamilton-Davies

**Affiliations:** 1 Queen Mary University London, London, UK; 2 University College London, London, UK; 3 The Royal Brompton & Harefield NHS Trust, London, UK; 4 Institute for Cardiovascular Sciences, University College London, London, UK; 5 Karolinska Institute, Stockholm, Sweden; 6 Department for Peri-operative Medicine, St Bartholomew’s Hospital, First floor, KGV Building, West Smithfield, London, UK

**Keywords:** Cardiac surgical procedures, Core endotoxin, Immunity, Sepsis, Staphylococcus

## Abstract

**Background:**

Morbidity and mortality following cardiac valve surgery is high. Immunity is an important contributor to outcome. This study examines the relationship of staphylococcal and endotoxin antibody levels to outcome following cardiac surgery.

**Methods:**

Using enzyme-linked immunosorbent assays (ELISA), we measured pre-operative levels of antibodies to endotoxin core (EndoCAb); 3 common staphylococcal epitopes and varicella on saved serum of 60 adult patients scheduled to undergo elective primary surgical aortic valve replacement (AVR). Primary outcome measure was post-operative length of stay (LOS) in hospital with secondary outcomes being development of infective complications, length of stay on the intensive care unit (ICU) and 30-day mortality. Patients were quartiled according to antibody levels and outcomes compared between the quartile groups using Mann-Whitney tests for length of stay and Fisher’s test for development of infection.

**Results:**

Sixty patients (34 M, 26 F) were recruited with mean age 73 years (IQR 66–78), mean body mass index (BMI) 27.7 (IQR 25–31) and EuroSCORE II 1.44 (0.95–1.99). Those patients in the lower quartile for pre-operative antibody level had a longer post-operative stay than the upper quartile. EndoCAb (median IgG level Q1 42.2 MU/ml vs Q4 256 MU/ml) 9 vs 6 days, *p* = 0.025; alpha-toxin (median IgG level Q1 63 U vs Q4 558 U) 10 vs 7 days, *p* = 0.034; teichoic acid (median IgG level Q1 14 U vs Q4 419 U) 10 vs 8 days, *p* = 0.441; staphylococcal enterotoxin A (median IgG level Q1 55 U vs Q4 427 U) 9 vs 7 days, *p* = 0.865; varicella zoster (median IgG level Q1 1.325 U vs Q4 2.54 U) 8 vs 7 days, *p* = 1.0; and combined antibody levels 10 vs 6 days, *p* = 0.017. There were no differences in the number developing post-operative infections for each antibody type. The combined antibody analysis suggested a reduction in proportion of individuals developing infection from the upper vs lower quartile: 0 vs 0.33, *p* = 0.042.

**Conclusions:**

This study again suggests the inverse relationship between endotoxin core antibody levels and outcome following aortic valve surgery as well as suggesting a similar relationship with antibodies to staphylococcus. There is no such relationship for antibody levels against an organism not providing a peri-operative threat. Understanding this relationship may enable therapeutic manipulation of immune status, re-evaluation of risk and further investigation of the low immune state.

**Trial registration:**

The patients in this study are a sub-group of the RELIEF AS study.

ClinicalTrials.gov identifier NCT02174471.

## Background

The risks of undergoing surgical replacement of the aortic valve are not inconsiderable with a mortality of up to 5.6% (Kvidal et al., 2000), and a high morbidity burden (Auensen et al., 2017).

Patients who experience complications following cardiac surgery may well have prolonged recovery periods as well as longer stay in hospital (Brown et al., 2008).

The risk factors for developing complications and early mortality have been well described and commonly used risk scoring systems (e.g. Parsonnet, EuroSCORE, EuroSCORE 2 (Parsonnet et al., 1989; Nashef et al., 2012; Nashef et al., 1999)) highlight these when used to predict the risk of adverse outcomes (Wang et al., 2014). Around 1–2% of patients undergoing cardiac surgery experience infective complications with staphylococcal organisms, and most of these do not have any of the known risk factors (Kanafani et al., 2009). Little, if anything, is known regarding the competence of the immune system in these patients. Do those who experience adverse outcomes have impaired immunity pre-operatively?

The relationship between diminished pre-operative immunity to endotoxin and post-operative complications was described over 20 years ago both in patients undergoing coronary artery bypass grafts (CABG) and in a separate study on patients undergoing cardiac valve replacement surgery (Bennett-Guerrero et al., 1997; Hamilton-Davies et al., 1997).

It is possible that the inverse relationship between pre-operative antibody levels and outcome following cardiac surgery may pertain to all peri-operative antigenic/infective threats. Identifying factors that predispose or protect against post-operative infection may allow mitigation of that risk.

It is important to understand the underlying relationship between pre-operative staphylococcal antibody levels and post-operative outcome before hypothesising and testing whether the pre-operative levels can be altered and whether this subsequently leads to an improved outcome.

In this study, we aimed to determine if the relationship between pre-operative anti-staphylococcal antibodies and outcome in patients undergoing aortic valve replacement was similar to that previously seen with pre-operative EndoCAb levels. In addition, it was intended to reconfirm the EndoCAb relationship with outcome and examine these relationships with an antibody not thought to be a threat in the peri-operative period, varicella-zoster virus (VZV).

## Methods

### Patient selection

The patients in this study were a convenience sample as a sub-group of a larger study (NCT02174471) looking at myocardial structure of patients undergoing aortic valve replacement with or without additional procedures.

Ethical approval for this study was given by the NRES Committee, London (Ref No 07/H0715/101).

Patients scheduled for aortic valve replacement were approached and verbal and written consent to participate was obtained from those willing to take part in the study. No individual identifiable data is included in this submission. We aimed to include all patients from the parent study who had undergone first-time isolated AVR.

Exclusion criteria were age under 18 years, recent or ongoing infection, pregnancy, an immunosuppressive condition and concomitant use of immunosuppressive therapy.

Following recruitment, prior to surgery, the patient was venesected and blood samples centrifuged for 15 min at 3000 rpm and the serum stored in cryotubes (Thermoscientific, Mass) at − 80 °C for later analysis.

We chose to assay for antibodies to the extracellular polypeptides alpha-toxin (AT) and staphylococcal enterotoxin A (SEA), and to teichoic acid (TA), a major surface antigen of the staphylococcal organism; all are present in almost all strains of *Staphylococcus aureus*. A high proportion (86–95%) of the *S. aureus* isolates in clinical infections produce an anti-alpha-toxin antibody response (Granstrom et al., 1983a). In cases of serious staphylococcal infection, the levels of alpha-toxin have been demonstrated to be very high, suggesting that the antigen is highly immunogenic (Colque-Navarro et al., 1993). Teichoic acid is particularly expressed in case of long-standing staphylococcal infection, for example, deep-seated wound infection or endocarditis (Colque-Navarro et al., 1998). It was also decided to assay for SEA antibodies as this toxin is the most commonly produced enterotoxin in *S. aureus* strains (Kanclerski et al., 1996). These three antibodies are likely to be reliably expressed in those patients undergoing cardiac surgery that may go on to develop staphylococcal infections.

### Antibody analysis

#### Anti-staphylococcal antibody analysis

The ELISA procedure for assaying alpha-toxin, teichoic acid, and staphylococcal enterotoxin A antibody levels has been detailed previously (Granstrom et al., 1983b; Colque-Navarro et al., 2000). Briefly, coating doses for the 96-well microtitration plates (Dynatech M-129B, Plochingen, Germany) with alpha-toxin, teichoic acid and SEA were established at 2.5 μg/mL, 1 μg/mL and 0.5 μg/mL, respectively. The working volume throughout the tests was 100 μL/well. The microtitration plates were coated with antigens diluted in phosphate-buffered saline (PBS), pH 7.4, and incubated overnight at 22 °C. The plates were washed and a serum dilution in PBS with Tween-20 0.05% *v*/*v* (PBS-T) of 1 in 1000 for α-toxin and SEA and 1 in 10,000 for teichoic acid was added to two coated wells. Positive and negative controls were included in each plate. The plates were incubated for 1 h at room temperature (20 °C). After washing the plates, alkaline phosphatase-conjugated goat anti-human antibody (Sigma) diluted in PBS-T was added to each well, and the plates were incubated for 2 h at room temperature. After the final wash, p-nitrophenyl- phosphate substrate (Sigma) was added. Titres were read when the positive controls reached previously established values at 405 nm on a Titertek Multiskan (Flow Laboratories, Irvine, Scotland) instrument.

The antibody levels were expressed as arbitrary units by using the reference line unit calculation method (Reizenstein et al., 1995).

#### EndoCAb analysis

Serum EndoCAb levels were measured with an ELISA using equimolar amounts of a lipopolysaccharide (LPS) from each of a selected *Escherichia coli*, *Salmonella typhimurium*, *Klebsiella aerogenes*, *Pseudomonas aeruginosa* rough mutant strain, lacking the LPS O-polysaccharide and part of the LPS outer core, but retaining the inner core structure. These were each complexed to polymyxin B, mixed in a cocktail in carbonate-bicarbonate buffer (pH 9.6), and the cocktail coated on 96-well polystyrene microtitre plates selected for optimal EndoCAb ELISA characteristics. Results are expressed as median units (MU) per millilitre, where 100 MU/mL is the median value of a population of 1000 healthy volunteers.

Test and control samples were diluted 1:200 with dilution buffer, and 100 μl of each sample to be assayed was added in triplicate to the wells of a pre-coated microtitre plate. The assay was standardised using a calibrated pooled-serum standard of a predetermined EndoCAb IgG concentration. An eight-point standard curve was constructed using doubling dilutions of the calibrated serum, which gave a range of IgG EndoCAb of 12.25 to 784 MU, and 100 μl of each added in triplicate to the microtitre plate. Following the addition of the samples and standards, the plate was covered and incubated for 1 h at 37 °C.

The plates were then washed three times with phosphate-buffered saline solution-polysorbate (Tween) buffer (sodium chloride, 0.138 M; phosphate, 0.01 M; pH 7.4 containing 0.10% [*v*/*v*] polyoxyethylene sorbitan monolaurate [Tween 20]) and finally blotted dry.

An alkaline phosphatase goat anti-human IgG antibody (Sigma-Aldrich Chemical Co; Poole, UK) was diluted 1:500 with dilution buffer, and 100 μl of conjugated antibody was added to each well; the plates were covered and incubated for 1 h at 37 °C.

After incubation, the plates were washed three times with Tween buffer. Substrate (100 μl), comprising 1 mg/mL disodium p-nitrophenyl phosphate in 0.05 M sodium bicarbonate; 0.05 M sodium carbonate 2:1 (*v*/*v*) containing 0.002 M magnesium chloride, was added to each plate and incubated at room temperature in the dark for approximately 30 min.

The plate was then read at 405-nm wavelength using an automated plate reader (Anthos Labtech; Salzburg, Austria). A standard curve was constructed at 405 nm against EndoCAb concentration, and the result of test samples was deduced from the curve. Results were rejected if the EndoCAb concentration of the control sample varied > 1 SD from its assigned value or the correlation coefficient for the standard curve was < 0.98.

#### VZV antibody analysis

These were assayed using a semi-quantitative test which enabled the positioning of the antibody levels relative to the other samples being tested (Biomerieux, Basingstoke, UK). The assay principle combines a two-step enzyme immunoassay sandwich method with a final fluorescent detection––enzyme-linked fluorescent assay (ELFA).

The solid-phase receptacle (SPR) acts as the solid phase, as well as the pipetting device. The reagents are ready-to-use and dispensed in the sealed reagent strips. The instrument performs all of the assay steps automatically, with the reaction medium being cycled in and out of the SPR several times.

After a preliminary wash step and dilution in the sample diluent, the sample is incubated in the SPR. Any anti-VZV antibodies present in the sample will bind to the VZV antigen coating on the interior of the SPR. Unbound components are removed in the subsequent wash step. Anti-human IgG antibodies conjugated with alkaline phosphatase will bind to any human IgG bound to the interior of the SPR. Washing removes unbound conjugate. During the final detection step, the substrate (4-methyl-umbelliferyl phosphate) is cycled in and out of the SPR. The bound conjugate enzyme present catalyses the hydrolysis of the substrate into a fluorescent product (4-methyl-umbelliferone), which is measured at 450 nm. The intensity of the fluorescence is proportional to the concentration of anti-VZV IgG antibodies in the sample. This value is automatically compared to the calibration curve and a result printed.

The result for each sample is expressed as the ‘test value’ (TV), a ratio of the ‘relative fluorescence value’ (RFV) of the sample to that of the stored value of the standard.

$$ \mathrm{TV}=\frac{\mathrm{RFV}\kern0.5em \mathrm{of}\kern0.5em \mathrm{sample}}{\mathrm{RFV}\kern0.5em \mathrm{of}\kern0.5em \mathrm{standard}\kern0.5em \mathrm{stored}\kern0.5em \mathrm{on}\kern0.5em \mathrm{system}} $$

#### Pre-operative risk scoring

EuroSCORE 2 values were obtained for each patient along with demographic information including age, gender, height, weight, body mass index, left ventricular ejection fraction, peak gradient across the aortic valve, presence of diabetes, presence of hypertension, NYHA class and glomerular filtration rate. All patients underwent routine screening prior to surgery for evidence of colonisation with methicillin resistant *Staphylococcus aureus*.

Diabetes was considered present if the patient had a pre-operative requirement for oral hypoglycaemic or insulin therapy. Left ventricular ejection fraction (LVEF), peak gradient across the aortic valve and estimated aortic valve area (AVA) were determined by standard 2D echocardiography performed within 6 weeks prior to surgery.

#### Outcomes

The primary outcome measure was post-operative length of stay in hospital. Secondary outcomes included length of stay on ICU, development of infection and mortality at 30 days. Peri-operative outcome data was collected regarding length of cardiopulmonary bypass, aortic close-clamp time, length of stay on the ICU, and on the development of infection, as well as survival up to 30 days. We used the Centre for Disease Control and Prevention (CDC) and National Healthcare Safety Network (NHSN) guidance to define the presence of a healthcare-associated infection (HAI) (Horan et al., 2008). Attributing whether or not there was a significant post-operative infection was determined by examining the following characteristics: We noted for all patients the clinical judgement by staff directly caring for the patient, direct observation of patients and wounds, and blood and microbiology laboratory results, whether samples were sent for culture of pathogenic organisms, whether any positive result was obtained, whether an antimicrobial agent was started and whether the clinical teams determined that there was a (HAI).

All patients received standard institutional antibiotic prophylaxis consisting of 1 g flucloxacillin and 1.5 mg/kg gentamicin.

#### Statistical analysis

Data are presented as absolute numbers and percentages for categorical variables and median and interquartile range for continuous variables. Continuous variables were compared by *t* tests and Mann-Whitney *U* tests whereas dichotomous variables were compared using Fisher’s exact test. Probabilities are two-tailed.

Given an average length of post-operative stay in our institution for uncomplicated valve surgery of 7 days, and a stay of 10 days or greater being considered prolonged (Bennett-Guerrero et al., 1997), we estimated that, at 80% power, with a 5% type II error rate, a sample size of 21 in each group would be sufficient to detect a 3-day increase in length of stay. This would require us to examine 84 patients in order to have sufficient numbers in each quartile.

After losing 32 patients from the initial cohort, we performed post hoc power calculations based on the demonstrated reduction in length of stay from 10 to 6 days. With 15 subjects in each quartile group, we are able to detect the change in length of stay with 80% power and 5% type II error rate. In a previous study comparing pre-operative EndoCAb levels, significant differences in outcome were demonstrated in a cohort of 60 patients (Hamilton-Davies et al., 1997).

## Results

A total of 149 patients were recruited in the parent trial, and 94 of these had isolated AVR. Sixty of these who underwent isolated first-time AVR had complete data with respect to available serum, blood analysis results and post-operative outcome information. Thirty-four patients did not have completed analysis of serum. There were no exclusions of recruited patients with complete data from the analysis.

Pre-operative characteristics and relevant intra-operative variables of the study population are presented in Table 1.

**Table 1:** Subject characteristics

Number	60
Age (years)	73 (66–78)
Male (%)	34 (57)
BMI (kg m^−2^)	27.7 (24.6–31.3)
LVEF (%)	73 (66–79)
Aortic Valve Peak gradient (mmHg)	75 (63–88)
Diabetes mellitus (%)	11 (18)
Hypertension (%)	49 (82)
NYHA	
Class I	10 (17)
Class II	34 (57)
Class III	14 (23)
Class IV	2 (3)
GFR (ml min^−1^)	72 (56–88)
EuroSCORE II	1.44 (0.95–1.99)
CPB time (min)	81 (71–106)
Aortic cross clamp time (min)	60 (53–73)

Data are presented as number of patients and percentage within the group or as median and interquartile range

*BMI* body mass index, *LVEF* left ventricular ejection fraction, *NYHA* New York Heart Association, *GFR* glomerular filtration rate, *CPB* cardiopulmonary bypass time

Patients with lower pre-operative antibody levels appeared to have a longer post-operative stay following aortic valve replacement.

**Fig. 1:**
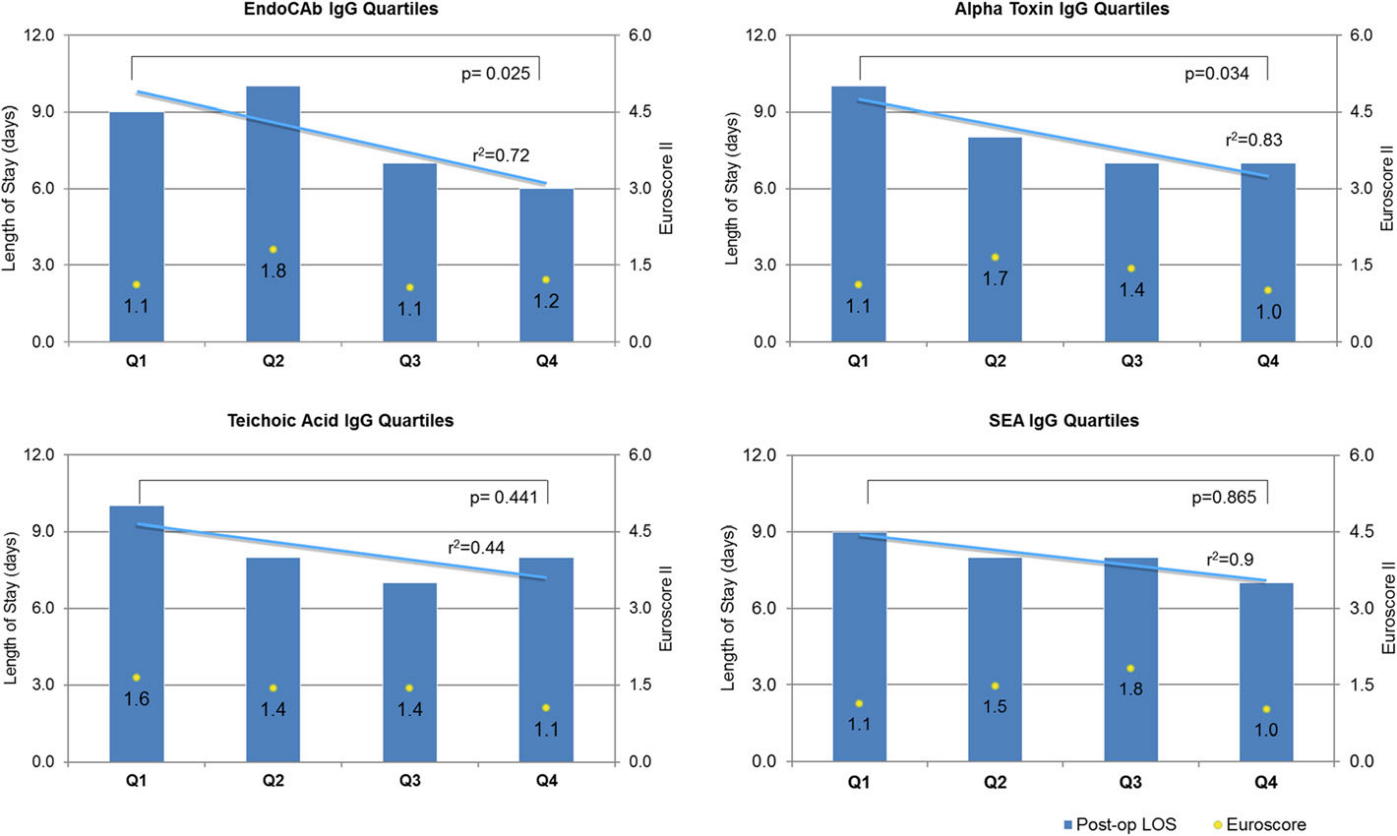
Patient cohort divided into quartiles for each measured antibody level. The median antibody level for each quartile is given. Median length of post-operative stay for each quartile (bars) and trend-line (blue line) and the median EuroSCORE II (number, yellow dot) is displayed for each group. (Q1––quartile 1, etc.)

**Fig. 2:**
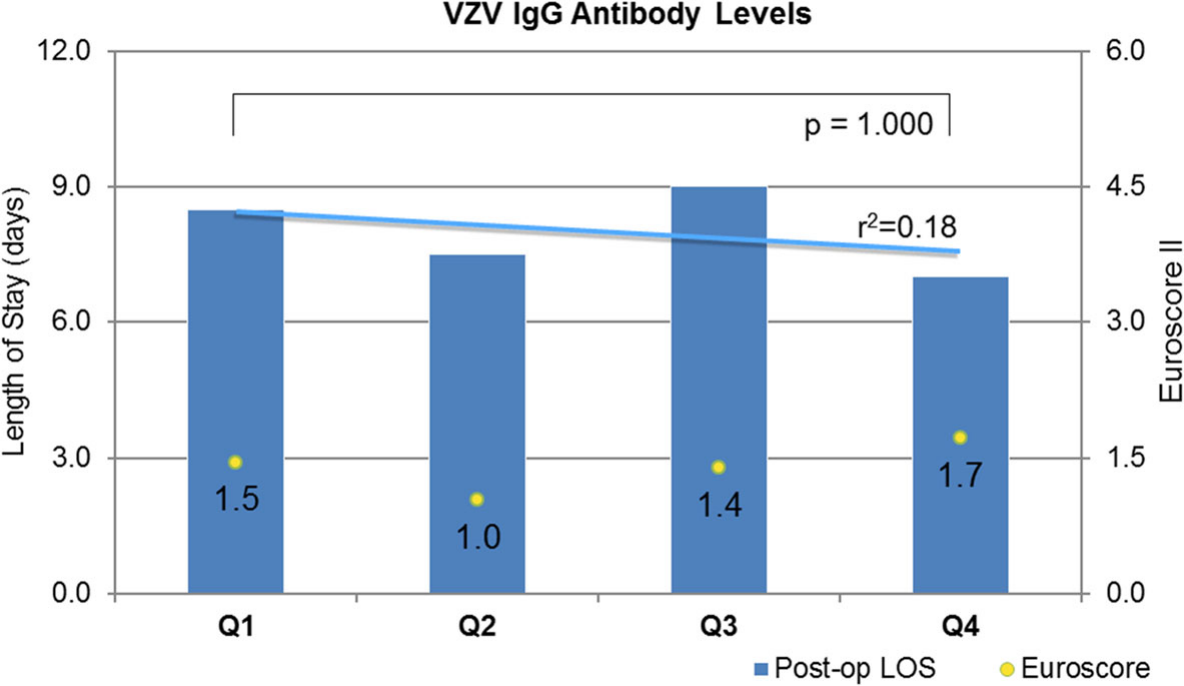
Patient cohort divided into quartiles for measured antibody level to varicella-zoster virus (VZV). The median antibody level for each quartile is given. Median length of post-operative stay for each quartile (bars) and trend-line (blue line) and the median EuroSCORE II (number, yellow dot) is displayed for each group. (Q1––quartile 1, etc.)

Post-operative outcome data is presented in Table 2. The median (IQ range) post-operative length of stay for the whole group was 8 (Nashef et al., 1999; Wang et al., 2014; Kanafani et al., 2009; Bennett-Guerrero et al., 1997; Hamilton-Davies et al., 1997) days, and there were no in-hospital deaths. One patient died 13 days following discharge from hospital. The relationship between antibody levels to endotoxin core and to the three staphylococcal epitopes and outcomes are given in Fig. 1. The relationship between VZV antibodies and outcomes are given in Fig. 2.

**Table 2:** Outcome characteristics

Number	60
Length of stay in ICU (days)	2.5 (2–4)
Length of post-operative stay in hospital (days)	8 (6–10)
Development of significant infection	14 (23)
Death (in hospital)	0
Death (within 30 days)	1 (1.7)

Data are presented as number of patients and percentage within the group or as median and interquartile range

*ICU* intensive care unit

**Fig. 3:**
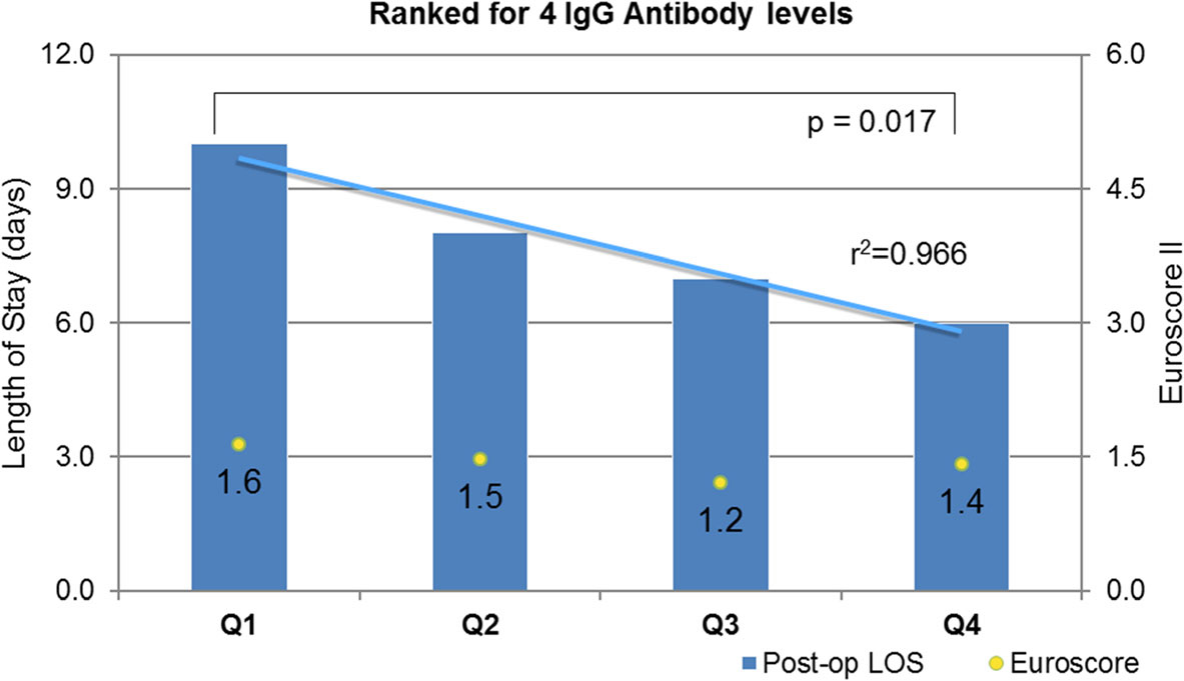
Patient cohort divided into quartiles for combined, ranked antibody levels for the four antibodies. Median length of post-operative stay for each quartile (bars) and trend-line (blue line) and the median EuroSCORE II (number, yellow dot) is displayed for each group. (Q1––quartile 1, etc.)

For each antibody assay the results for all patients were ranked in ascending order from 1 to 60; thus, each participant had an indication of the relative ranking of each antibody level result ranging from 1 to 60. The antibody level rankings for the EndoCAb and the three anti-staphylococcal assays were added to create a cumulative antibody level ranking for each patient. The group of 60 patients were then divided into quartiles based on the cumulative ranking of antibody levels. Figure 3 shows the length of stay and EuroSCORE II for each quartile within this group. The median values for various demographic parameters for each quartile of the ranked group are shown in Table 3.

**Table 3:** Outcome characteristics for the combined rank group

	Q1	Q2	Q3	Q4	Q1 vs Q4
Number	15	15	15	15	
Age (years)	74	71	75	70	*p* = 0.289
BMI (kg m^−2^)	26.2	26.9	25.7	28.1	*p* = 0.342
LVEF (%)	73	72	77	69	*p* = 0.908
EuroSCORE II	1.64	1.47	1.21	1.42	*p* = 0.429
CPB time (min)	85	77	78	89	*p* = 0.392
Aortic cross clamp time (min)	59	58	61	62	*p* = 0.313
Length of stay in ICU (days)	3	4	2	2	*p* = 0.348
Post-operative stay (days)	10	8	7	6	*p* = 0.017
Proportion with infection	0.33	0.33	0.27	0	*p* = 0.042
Death	0	0	1	0	

Data are presented as median for each quartile (Q1–Q4). The proportion with infection is displayed as the proportion of the quartile group who developed an infection

*BMI* body mass index, *LVEF* left ventricular ejection fraction

Fourteen patients were deemed as having a clinically significant infection (median LOS 11 days) compared with the 46 patients who were not deemed to have infection (median LOS 7 days) (*p* = 0.0033). Three of the 14 returned positive microbiology cultures (1 *Staphylococcus aureus* wound infection, 1 *Staphylococcus epidermidis* wound infection and one *Escherichia coli* wound infection). The remaining 11 patients with no positive microbiology culture result had abnormal CRP and WCC profiles along with clinical suspicion of infection and were each started on antibiotic therapy. In total, 6 patients developed a wound infection of which one was a primary deep incisional infection and the remaining were superficial infections. Eight patients developed a chest infection (PNU1 type). We were unable to determine a relationship between individual antibody levels and the likelihood of developing a clinically significant infection. However, with the analysis of combined antibody group, there appeared to be an association between pre-operative antibody levels and the likelihood of developing a post-operative HAI (Table 4).

**Table 4:** Percentage within each quartile for each antibody type and for the ranked antibody group that developed a clinically significant infection

	Q1	Q2	Q3	Q4	
Number	15	15	15	15	
Teichoic acid	33.3%	26.7%	13.3%	20%	*p* = 0.682
Alpha-toxin	40%	20%	13.3%	20%	*p* = 0.427
Staphylococcal enterotoxin A	13.3%	20%	46.7%	13.3%	*p* = 1.000
EndoCAb	26.7%	33.3%	6.7%	26.7%	*p* = 1.000
4-way rank	33%	33.3%	26.7%	0%	*p* = 0.042

## Discussion

The results from this study suggest a relationship between pre-operative staphylococcal antibody levels and outcome following aortic valve surgery. This result appears to be similar to previously published relationships between EndoCAb levels and outcome after cardiac surgery. Our study reconfirms that relationship between pre-operative EndoCAb levels and length of stay following cardiac surgery.

Several studies have now demonstrated the link between pre-operative immune status to endotoxin and outcome following major surgery (Bennett-Guerrero et al., 1997; Hamilton-Davies et al., 1997; Bennett-Guerrero et al., 2001). Endotoxin exposure is common during cardiopulmonary bypass (CPB) (Andersen et al., 1987) and may be due to translocation across the bowel as a consequence of episodes of presumed hypo-perfusion. The protective ability of antibodies to this may not be surprising, yet the innate immunity of the pre-operative patient is not routinely explored, and is not incorporated in standard risk assessment algorithms. Endotoxin and Gram-negative organisms are not the only peri-operative threat that patients undergoing CPB are potentially exposed to in the peri-operative phase with staphylococcal wound infection complicating some 3–4% of cardiac procedures (Fowler Jr. et al., 2005). A large study carried out in the USA on over 8000 patients undergoing cardiopulmonary bypass surgery found an overall mortality rate of 4% compared with over 17% for those developing post-operative MRSA (Allen et al., 2014). The economic implications of cardiac surgical patients developing staphylococcal bacteraemia is great with a three-fold increase in cost for the group developing infective complications (Brown et al., 2008; Coskun et al., 2005).

The variability in anti-staphylococcal antibodies in healthy volunteers and in pre-operative surgical patients has been demonstrated (Moravcova et al., 2016; Colque-Navarro et al., 2010) and that variability in the surgical patients appears to be greater than that in a healthy volunteer population (Moravcova et al., 2016).

Much work has been performed on antibodies to various staphylococcal epitopes and, in particular, how these levels are related to certain infective or inflammatory clinical situations (Kanclerski et al., 1996). Antibodies to alpha-toxin and teichoic acid have been shown to relate to development of complications during staphylococcal septicaemia with lower initial levels relating to a poorer antibody response (Colque-Navarro et al., 1998).

Not only is staphylococcus the major organism responsible for cardiac surgical site infections (SSIs), it has also been shown that the alpha-toxin fragments secreted by staphylococcus can impair gut mucosal integrity (Kwak et al., 2012). This may further worsen the potential for sepsis by enabling Gram-negative organism or endotoxin translocation from the gut lumen to the bloodstream (Swank & Deitch, 1996).

As with previous EndoCAb studies, this study has demonstrated that an inverse outcome relationship also exists for antibody levels to staphylococcus, another peri-operative threat. A relationship could not be established with antibody levels to varicella, a threat not encountered in the operative environment. This raises a question as to whether the outcome relationship only holds for antibodies to peri-operative threats. It is possible that some patients have an impaired immunological ability to react to these encountered threats and, when they are challenged, are unable to mount a suitable immune response resulting in adverse post-operative recovery.

The patients with low levels of several antibody types appear to have worse outcomes than those with low levels in only one type or higher levels in all types.

Our study suggests that pre-operative immunity may impact on post-operative outcome. Current commonly used risk scoring methods do not take any account of immune competence (Nashef et al., 2012). If immunity is important, then it follows that an assessment of immune system competence should be incorporated into those risk stratification processes to allow more accurate risk profiling and consequently a more informed discussion with the patient whilst considering surgical options for heart disease.

It is important to note that these conclusions are being drawn from observational data collected from a relatively small number of patients with a moderate degree of variability in the pre-operative characteristics. Due to the small study size, little conclusion can be drawn as to whether developing infectious complications is associated with antibody levels. Moreover, antimicrobial therapy is often started empirically, before culture results are known, confounding the standardised definitions of presence of infection. We have no information regarding immune responsiveness at the cellular level and are making an assumption that pre-operative antibody levels reflect immune responsiveness at some level. Immune responsiveness is a dynamic process, and we are measuring antibody levels at one time point. The staphylococcal antibodies that we chose to study have previously been associated with outcome studies, but there many epitopes that are as yet unexplored and which may have a greater or lesser contribution to effects on outcome. We chose to use post-operative length of stay (LOS) as the main measure of outcome in this study in order to allow comparison with similar previously published work. LOS is readily understood by patients and clinicians and reflects a composite outcome accounting for multiple post-operative complications. Other non-clinical problems may well impact on LOS (e.g. failure to reach adequate anticoagulation, social care needs), and there may be benefit for future studies to explore the specific morbidity domains that impact on LOS, e.g. CPOMS (Sanders et al., 2012).

If the patient is thought to be at elevated risk of adverse outcome as a consequence of impaired immunity, is there an alternate treatment option that has less risk? It is not yet known whether transcatheter procedures, for example transcatheter aortic valve replacement (TAVR) (Moat et al., 2011), expose the patient to the same level of antigenic threat compared to open surgery. It may be that a TAVR is a preferred option for some patients judged to be at higher risk because of their immune system performance.

Can the risk conferred by impaired immune performance be altered? The association of low levels of antibody to both staphylococcus and endotoxin may indicate that pre-operative elevation of these could be a potential therapeutic strategy and this might be enabled by either active or passive immunisation. We have previously demonstrated that pre-operative EndoCAb elevation is possible both actively and passively (Hamilton-Davies, 1998; Hamilton-Davies et al., 1996). Active vaccination may offer some benefit; however, it is likely to be a less effective strategy in a patient who has an impaired immune response.

Anti-endotoxin therapies have been tried in sepsis and failed to consistently show benefit in clinical trials (McCloskey et al., 1994; Bone et al., 1995). One question arising from these studies has been whether the products are being correctly targeted at the patient population groups. Treatment in sepsis at time zero is often not feasible, and the underlying immune status of the treatment group is not known. Major elective surgery with a reasonably high-mortality/morbidity outcome profile would seem to allow for a much more controlled study environment and is also the originating population for a proportion of those developing sepsis and multiple organ failure (MOF). Surgical site infection (SSI) following cardiac surgery is often a primary negative event that precipitates a progression of events terminating in MOF and death (Allen et al., 2014). The major infective elements contributing to SSIs are represented by staphylococcal organisms and Gram-negative organisms (up to 35%) containing the pro-inflammatory components of the endotoxin core (Soderquist, 2007; Garey et al., 2006). These may also contribute to the development of post-operative ventilator-associated pneumonia (VAP) (Hortal et al., 2009; Kollef et al., 1997).

Targeting of potential therapeutic agents towards those that can be shown to be ‘at risk’ due to lower pre-operative levels of protective antibodies against endotoxin core and staphylococcal epitopes along with other conventional risk criteria may enable a clearer assessment as to their efficacy.

It is currently not known if the antibody levels measured pre-operatively are temporarily low or chronically low due to a hypo-responsive immune system and whether it is chronic immune hypo-responsiveness that is the real issue associated with a poor outcome. It is possible that the chronic immune hypo-responsiveness may be genetically determined; and thus, these patients may not be expected to benefit from active immunisation. It may be that they would fall into the same population of patients that are observed to succumb to secondary sepsis (van Vught et al., 2016) and that have been linked to the genetic signature associated with endotoxin tolerance (Pena et al., 2014; Cavaillon & Adib-Conquy, 2006). Determining the answer to these questions will allow a more precise definition of the at risk group and, in combination with other conventional risk scoring systems incorporating oxygen delivery profiles, will probably lead to a more comprehensive risk analysis.

## Conclusion

This study suggests an apparent association between pre-operative antibody levels and outcome following cardiac surgery. An inverse relationship is seen between outcome following AVR and pre-operative antibody levels to staphylococcus and endotoxin, important peri-operative threats. There is no such relationship for antibody levels against an organism not thought to pose a peri-operative threat.

The result supports our assertion that this area deserves further exploration and investment. Understanding this relationship may enable therapeutic manipulation of immune status, re-evaluation of peri-operative risk and further investigation of the low immune state.

## Abbreviations

AT: Alpha-toxin
AVA: Aortic valve area
AVR: Aortic valve replacement
BMI: Body mass index
CABG: Coronary artery bypass grafts
CDC: Centre for Disease Control and Prevention
CPB: Cardiopulmonary bypass
CPOMS: Cardiac postoperative morbidity score
CRP: C-reactive protein
ELFA: Enzyme-linked fluorescent assay
ELISA: Enzyme-linked immunosorbent assay
EndoCAb: Endotoxin core antibody
HAI: Healthcare-associated infection
ICU: Intensive care unit
IQR: Interquartile range
LOS: Length of stay
LPS: Lipopolysaccharide
LVEF: Left ventricular ejection fraction
MOF: Multiple organ failure
MRSA: Methicillin resistant *Staphylococcus aureus*
NHSN: National Healthcare Safety Network
NYHA: New York Heart Association
RFV: Relative fluorescence value
SEA: Staphylococcal enterotoxin A
SPR: Solid phase receptacle
SSI: Surgical site infection
TA: Teichoic acid
TAVR: Transcatheter aortic valve replacement
TV: Test value
VAP: Ventilator-associated pneumonia
VZV: Varicella-zoster virus
WCC: White cell count
